# Mesenchymal Stem Cells Transfer Mitochondria to the Cells with Virtually No Mitochondrial Function but Not with Pathogenic mtDNA Mutations

**DOI:** 10.1371/journal.pone.0032778

**Published:** 2012-03-06

**Authors:** Young Min Cho, Ju Han Kim, Mingoo Kim, Su Jin Park, Sang Hyeok Koh, Hyo Seop Ahn, Gyeong Hoon Kang, Jung-Bin Lee, Kyong Soo Park, Hong Kyu Lee

**Affiliations:** 1 Department of Internal Medicine, Seoul National University College of Medicine, Seoul, South Korea; 2 Department of Biomedical Informatics, Seoul National University Biomedical Informatics (SNUBI) and Interdisciplinary Program of Medical Informatics, Seoul, South Korea; 3 Department of Pediatrics, Seoul National University College of Medicine, Seoul, South Korea; 4 Department of Pathology, Seoul National University College of Medicine, Seoul, South Korea; 5 Department of Forensic Medicine, Seoul National University College of Medicine, Seoul, South Korea; 6 Department of Internal Medicine, Eulji University College of Medicine, Seoul, South Korea; Instituto de Investigación Hospital 12 de Octubre, Spain

## Abstract

It has been reported that human mesenchymal stem cells (MSCs) can transfer mitochondria to the cells with severely compromised mitochondrial function. We tested whether the reported intercellular mitochondrial transfer could be replicated in different types of cells or under different experimental conditions, and tried to elucidate possible mechanism. Using biochemical selection methods, we found exponentially growing cells in restrictive media (uridine^−^ and bromodeoxyuridine [BrdU]^+^) during the coculture of MSCs (uridine-independent and BrdU-sensitive) and 143B-derived cells with severe mitochondrial dysfunction induced by either long-term ethidium bromide treatment or short-term rhodamine 6G (R6G) treatment (uridine-dependent but BrdU-resistant). The exponentially growing cells had nuclear DNA fingerprint patterns identical to 143B, and a sequence of mitochondrial DNA (mtDNA) identical to the MSCs. Since R6G causes rapid and irreversible damage to mitochondria without the removal of mtDNA, the mitochondrial function appears to be restored through a direct transfer of mitochondria rather than mtDNA alone. Conditioned media, which were prepared by treating mtDNA-less 143B ρ^0^ cells under uridine-free condition, induced increased chemotaxis in MSC, which was also supported by transcriptome analysis. Cytochalasin B, an inhibitor of chemotaxis and cytoskeletal assembly, blocked mitochondrial transfer phenomenon in the above condition. However, we could not find any evidence of mitochondrial transfer to the cells harboring human pathogenic mtDNA mutations (A3243G mutation or 4,977 bp deletion). Thus, the mitochondrial transfer is limited to the condition of a near total absence of mitochondrial function. Elucidation of the mechanism of mitochondrial transfer will help us create a potential cell therapy-based mitochondrial restoration or mitochondrial gene therapy for human diseases caused by mitochondrial dysfunction.

## Introduction

In the 1940s, Lederberg and Tatum discovered the conjugation phenomenon, which is a process of a unidirectional transfer of genetic information essential for survival on a minimal medium through the formation of a physical bridge between a donor bacterium and a recipient bacterium [1]. Similar to the earlier findings in prokaryotes, it was recently reported that human mesenchymal stem cells (MSCs) can transfer mitochondria (microorganelles containing their own genetic information) to the cells with nonfunctional mitochondria incapable of aerobic respiration due to defective or deleted mitochondrial DNA (mtDNA) [2]. However, the mechanism of intercellular mitochondrial transfer is still elusive. It was postulated that the MSC actively transfers mitochondria to the cells with severely compromised mitochondrial function through a tunneling, tube-like structure [2]. In addition, it is conceivable that the cells with defective mitochondrial function could uptake the vesicles containing mitochondria that budded off from the donor cells. However, isolated mitochondria or platelets, which contain mitochondria, could not restore mitochondrial function of the cells with nonfunctional mitochondria [2]. Furthermore, it is uncertain whether the restoration of mitochondrial function is mediated by the transfer of mtDNA alone, which is similar to the conjugation phenomenon, or by the transfer of intact mitochondrial particles.

In this study, firstly, we investigated whether the reported intercellular mitochondrial transfer [2] could be replicated in different types of cells or under different experimental conditions. In this regard, we used mtDNA-less ρ^0^ cells derived from human osteosarcoma 143B cells lacking thymidine kinase activity by long-term treatment with ethidium bromide (EtBr) [3]. Secondly, to address whether the restoration of mitochondrial function is mediated through a transfer of mtDNA alone or intact mitochondrial particles, we used rhodamine-6G (R6G), which causes abrupt and irreversible damage to the mitochondrial function without the removal of mtDNA [4], [5], [6], [7]. Finally, to explore the possibility of therapeutic implications, we examined whether the mitochondrial transfer could occur in the cells harboring mtDNA mutations relevant to human diseases, such as A to G substitution at np 3243 in tRNA^leu(UUR)^ (OMIM #540000 or #520000) and 4,977 bp large deletion (OMIM #530000).

## Materials and Methods

### Coculture experiments

The 143B ρ^+^ cell and the 143B ρ^0^ cell, which was established from the 143B ρ^+^ cell through long-term treatment with EtBr, were generous gifts from Professor Yau-Huei Wei from National Yang-Ming University in Taiwan. Since the 143B ρ^0^ cell is characterized by the absence of a functional respiratory chain, no thymidine kinase activity, and dependence on uridine and pyruvate [3], we grew the cells in Dulbecco's Modified Eagle's Media (DMEM, Invitrogen, Carlsbad, CA) supplemented with 100 µg/ml BrdU (Sigma, St. Louis, MO), 50 µg/ml uridine (Sigma), and 10% FBS. Southern blot analysis and PCR amplification of mtDNA target sequences confirmed the absence of any residual mtDNA (data not shown). Trans-mitochondrial cytoplasmic hybrids (or cybrids) harboring either the A3243G or 4,977 bp deletion mutation with varying degrees of mutational load (heteroplasmy rate) that shared an identical nuclear background with 143B, were also cultured in the same media. The cybrids with A3243G mutation were derived by fusion of 143B ρ^0^ cells with platelets from a patient harboring A3243G mutation, according to the standard protocol [8]. The cybrids with 4,977 bp deletion mutation were also gifts from Professor Wei.

**Figure 1:**
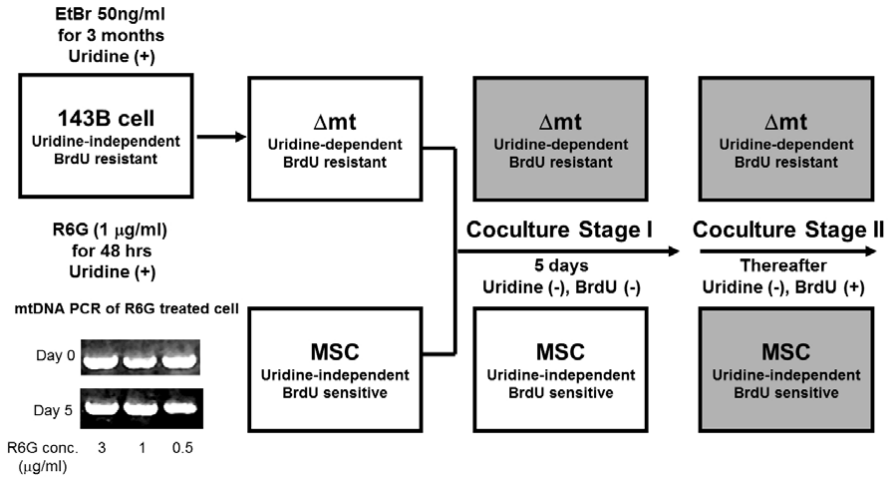
Scheme of coculture experiments. Mitochondrial dysfunction was induced by long-term treatment of ethidium bromide (EtBr), which is known to remove mtDNA while sparing nuclear DNA, or by short-term treatment of rhodamine 6G (R6G), which tightly binds to the mitochondrial inner membrane and destroys the mitochondrial respiratory function. Gel image shows that the mtDNA was still present until five days after R6G treatment (inset). We cocultured MSCs with 143B-derived cells with severely compromised mitochondrial function (Δmt) in uridine^−^ BrdU^−^ media for five days (Stage I) and in uridine^−^ BrdU^+^ media thereafter (Stage II). Cybrid cells harboring either the A3243G or 4,977 bp deletion mutation were also cultured in a similar way, with slight modification. For details, refer to the *Materials and Methods* section. The gray-colored box refers to the condition, which was expected to be fatal for the cells.

The schemes of the coculture procedures are shown in Fig. 1. We cocultured human bone marrow derived MSCs at 1.5×10^5^ cells with those cells listed above (i.e., 143B ρ^0^, cybrids with A3243G, and cybrids with 4,977 bp deletion) at 3×10^5^ cells in a 100 mm culture dish with DMEM supplemented with 10% FBS and 50 µg/ml uridine for 24 h. At this stage, we removed BrdU from the culture medium; we did not add pyruvate, since DMEM contains a sufficient amount of pyruvate (110 µg/ml). Then, we changed the culture medium with DMEM supplemented by 10% FBS, but without uridine (Stage I). After five days of coculture in the DMEM without uridine, we added 100 µg/ml BrdU to the same culture condition and continued cultivation for several weeks (Stage II). These experiments were repeated by adding cytochalasin B (5 µg/ml) (Sigma), an inhibitor of chemotaxis and cytoskeletal assembly.

For the quick removal of mitochondrial function, we treated 143B ρ^+^ cells with rhodamine 6G (R6G, Invitrogen) at 1 µg/ml for 48 h. After 48 h of treatment with 1 µg/ml of R6G, the 143B ρ^+^ cells could not survive in the restrictive media lacking uridine. To examine whether mtDNAs were removed after R6G treatment, we performed PCR analysis with genomic DNA extracted from the 143B ρ^+^ cells before and 5 days after R6G treatment. PCR amplification was performed using primers, 5′-CCACAACTCAACGGCTACAT-3′ (10131–10350) and 5′-CTGTGGGTGGTTGTGTTGAT-3′ (10851–10832). The PCR product (721 bp) was examined on 1.5% agarose gel. Using these R6G-treated cells, we repeated the above coculture procedures (Stage I and II) after careful washing with phosphate buffered saline (PBS).

### Nuclear DNA fingerprinting and mtDNA genotyping

Nuclear DNA fingerprinting was performed by using short tandem repeat (STR) loci in the AmpF/STR Identifiler™ PCR kit (Applied Biosystems, Foster, CA), as described previously [9]. We performed direct sequencing of mtDNA hypervariable regions (HVRs) using primers (L15751-H16439 for HVR I and L048-H922 for HVR II and HVR III) as described previously [10].

We also performed a PCR restriction fragment length polymorphism (RFLP) analysis for mtDNA polymorphisms. In this experiment, we used cybrid cells harboring mtDNA characterized by absence of both 10394 *Dde I* and 10397 *Alu I* sites, since the MSC used in this study had +10394 *Dde I* (A10398G variant) and +10397 *Alu I* (C10400T variant) sites. PCR amplification was performed using primers, 5′-CCACAACTCAACGGCTACAT-3′ (10131–10350) and 5′-CTGTGGGTGGTTGTGTTGAT-3′ (10851–10832). The PCR product (721 bp) was digested with *Dde I* and *Alu I* and the RFLP pattern was examined on 1.5% agarose gel.

### Measurement of intracellular ATP content

The cellular contents of ATP were measured using the luciferin-luciferase reaction with an ATP bioluminescence somatic cell assay kit (Sigma). The harvested cells, which were grown in culture media (DMEM with 10% FBS) containing uridine but not BrdU, were suspended with KRH buffer containing 0.1 mM glucose and 0.2% bovine serum albumin (BSA). These cell suspensions were incubated in a 37°C shaking water bath. Following the addition of an assay mixture containing luciferin and luciferase, luminescence was measured immediately in a bioluminometer equipped with an injector (Lumat LB 9507, Berthold, Bad Wildbad, Germany). The amounts of ATP were determined by running an internal standard, and they were adjusted for the amount of protein that was determined via the Bradford method.

### Measurement of oxygen consumption

Oxygen consumption was measured with a respirometer (Oxygraph-2k; Oroboros Instruments, Innsbruck, Austria). Standardized instrumental and chemical calibrations were performed to correct back-diffusion of Oxygen into the chamber from the various components. The suspension of cells in culture media (DMEM with 10% FBS) containing uridine but not BrdU was added to the respirometer with a concentration of 1.0×10^6^ cells/ml. The respirometer was operated at 37°C with a 2 ml volume. Oxygen flux was analyzed by the software provided by the manufacturer, which is capable of converting nonlinear changes in the negative time derivative of the oxygen concentration signal.

### Cell migration assay

The QCM™ 24-Well Colorimetric Cell Migration Assay kit (Chemicon, Temecula, CA), which has an 8-µm pore size polycarbonate membrane and is based on the Boyden chamber principle, was used for this experiment. The assay provides quantitative or colorimetric information on the amount of the cells, which migrated from the upper chamber to the lower chamber across the microporous membrane. Briefly, MSCs were prepared at 1.0×10^6^ cells/ml after 24 h treatment of serum-free DMEM. We added 500 µl of serum-free DMEM containing MSCs to the upper chamber in the presence of conditioned medium, which was prepared by treating 143B ρ^0^ cells, when the cells reached 60% confluence in 100 mm culture dish, with 10 ml of standard or uridine-free media for 48 h, in the lower chamber. Serum-free fresh DMEM was used as control. MSCs that had migrated through the polycarbonate membrane were incubated with cell-staining solution and were subsequently extracted and detected on a standard microplate reader at 560 nm according to the manufacturer's instructions.

### Electron microscope (EM)

Cultured cells were sequentially fixed with 2.5% glutaraldehyde and 1% OsO_4_. Thereafter we followed the standard procedures for EM examination. For the scanning EM, the samples were mounted on aluminum stubs with conductive paste, coated with a 20–30 nm thickness of platinum in an ion coater (Eiko IB-3, Eiko Engineering, Ibataki, Japan), and viewed in a scanning EM (S-520; Hitachi, Tokyo, Japan). For transmission EM examination, heavy metal staining was done with 4% uranyl acetate and lead citrate, and the samples were examined through H-7100 EM (Hitachi) at 50 kV.

### Microarray

Gene expression profiles of MSCs at 0, 6, 12, 24, and 48 h after treatment with conditioned media, which were obtained by treating 143B ρ^0^ cells with uridine-free media for 48 h. Microarray experiments were performed using GeneChip® Human Genome U133 Plus 2.0 array (Affymetrix, Santa Clara, CA), according to the manufacturer's protocol. For normalization, the Robust Multichip Average (RMA) was applied, which was implemented in BioConductor in the R statistical language [11]. All data is MIAME compliant and the raw data has been deposited in GEO (accession number: GSE33831).

### Statistical analysis

Data are shown as means ± SD. Statistical significance was evaluated by the Wilcoxon signed rank test, and *p*<0.05 was considered significant. For differential expression analysis, we used Bayes-ANOVA by Baldi et al. [12], which identifies genes whose expression levels differ between time points. In comparison to standard ANOVA, Bayes-ANOVA is more stable—especially with a small number of replicates—because it uses the posterior variance, which integrates data variance from a specific gene and prior variance from the neighboring genes in the Bayesian framework [12]. For multiple hypothesis testing correction, we used the Benjamini and Hochberg method to obtain the false discovery rate. For a cluster analysis of gene expression profiles, we used the self-organizing map (SOM) method implemented in BioConductor in the R statistical language. To visualize the global relationship of individual clusters, we performed a BioLattice analysis [13] with the clusters created by SOM. BioLattice enables a better biological interpretation of microarray gene expression data by organizing clusters into a unified lattice of concepts.

## Results

### Coculture of MSC with cells lacking mitochondrial function

**Figure 2:**
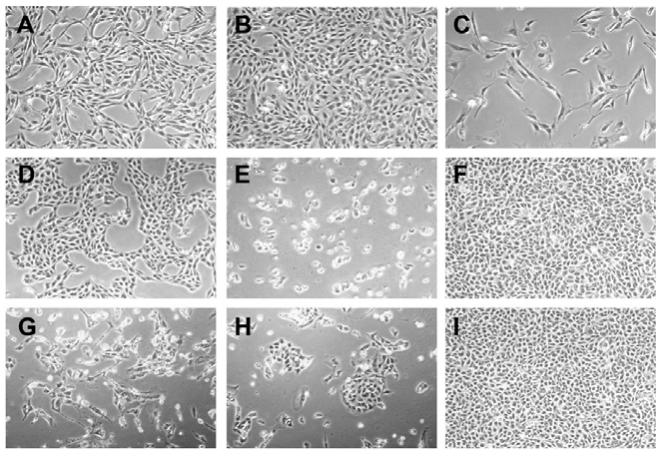
Characteristics and culture results of mesenchymal stem cells (MSCs) and 143B ρ^0^ cells. (A) MSCs in DMEM had a fibroblast-like appearance. (B) MSCs survived in uridine-free media, (C) but they were sensitive to BrdU. (D) The 143B ρ^0^ cells in permissive media supplemented with uridine had an epithelial cell-like appearance. (E) The 143B ρ^0^ cells could not survive in uridine-free media, (F) but they were resistant to BrdU treatment. (G) At day 4 of Stage II coculture in uridine^−^ BrdU^+^ media, fibroblast-like MSCs could not proliferate and gradually died with a senescent appearance. (H) After 7∼10 days in the uridine^−^ BrdU^+^ media, we observed multiple foci of colonizing cells, which had distinct epithelial-like morphology of 143B cells on microscopic examination; (I) thereafter, they grew exponentially to reach confluence. All are magnified at ×40.

The mtDNA-less 143B ρ^0^ cells have neither an electron transfer chain function nor thymidine kinase activity. Therefore, they are characterized by uridine dependence and BrdU resistance [3]. In contrast, MSCs from human bone marrow have normal mitochondrial function and thymidine kinase activity, and they are both uridine-independent and BrdU-sensitive. Firstly, we confirmed the difference in uridine dependence and BrdU sensitivity between 143B ρ^0^ and bone marrow-derived MSCs. As we expected, MSCs survived in uridine-free media but were sensitive to BrdU (Fig. 2 *A–C*), whereas 143B ρ^0^ cells could not survive in uridine-free media and were resistant to BrdU (Fig. 2 *D–F*). The scheme of the coculture procedures is shown in Fig. 1. The 143B ρ^0^ cells were cocultured with MSCs in permissive media with uridine supplementation for one day and then in restrictive media lacking uridine for five days (Stage I). After three days of coculture in restrictive media, most of the epithelial-like 143B ρ^0^ cells died, while fibroblast-like MSCs remained unaffected. After five days of coculture in restrictive media, we added BrdU to the media (Stage II). Thereafter, fibroblast-like MSCs could not proliferate and gradually died with a senescent appearance (Fig. 2 *G*). After 7∼10 days in the uridine^−^ BrdU^+^ media, we observed multiple foci of colonizing cells, each of which had a distinct epithelial-like morphology of 143B cells on microscopic examination (Fig. 2 *H*); they also grew exponentially to the confluence (Fig. 2 *I*).

The 143B ρ^+^ cells (parental cells of ρ^0^) treated with 1 µg/ml R6G for 48 h could not survive in the restrictive media without uridine supplementation (data not shown). However, mtDNA was still detected by PCR (shown as inset in Fig. 1). We added MSCs to the R6G-treated 143B ρ^+^ cells and repeated the experimental procedures in the same manner as was done for the 143B ρ^0^ cells. After 7∼10 days in the uridine^−^ BrdU^+^ media, we also found multiple foci of exponentially colonizing cells with epithelial-like morphology (data not shown). The addition of cytochalasin B, an inhibitor of chemotaxis and cytoskeletal assembly, yielded no exponentially growing colonies in coculture experiments using either 143B ρ^0^ or R6G-treated 143B ρ^+^ cells (data not shown).

### Genetic analysis of recuperated cells

**Figure 3:**
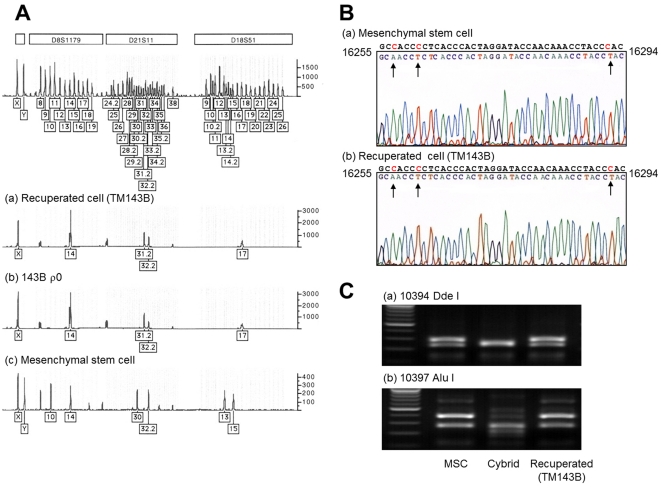
Genetic analysis of rapidly proliferating cells survived after coculture. (A) The cells survived after coculture showed genetic identities with 143B ρ^0^ cell nuclei. The boxed numbers and corresponding peaks represent locations of polymorphisms for each short tandem-repeat marker. (B) Since ρ^0^ cells did not have any detectable mtDNA, the mtDNA of the cells survived after coculture came solely from the MSCs. The hypervariable region sequences of the cells were identical with the MSCs. The arrows indicate sequence variations compared to the Cambridge reference sequence. (C) PCR-RFLP for 10394 *Dde I* and 10397 *Alu I* of mtDNA. The recuperated cells in the coculture experiment with R6G treatment to a cybrid harboring mtDNA with +10394 *Dde I* and +10397 *Alu I* were revealed to have mtDNAs with both −10394 *Dde I* and −10397 *Alu I*, both of which were identical to the MSCs.

Normally, none of 143B ρ^0^, R6G-treated 143B ρ^+^, or MSC could survive in uridine^−^ BrdU^+^ media; therefore, the exponentially growing cells in uridine^−^ BrdU^+^ media must be the cells with normal mitochondrial function but with no thymidine kinase activity—characters that could be obtained through the exchange of genetic materials between cocultured cells. Nuclear DNA fingerprints showed that the cells that survived in uridine^−^ BrdU^+^ media in both coculture conditions had the same genetic identities as the 143B cells (Fig. 3 *A*). More detailed data on DNA fingerprinting are shown in Table S1. On the other hand, the mtDNA hypervariable region sequences of those cells were completely identical to those of the MSCs from both coculture experiments of 143B ρ^0^ with MSCs (Fig. 3 *B* and Table S2) and R6G-treated 143B ρ^+^ cells with MSCs (data not shown). To further address whether mtDNAs from the MSCs replaced the mtDNAs of R6G-treated cells, we repeated the coculture experiment with R6G treatment, with a cybrid harboring 143B nuclear genome and mtDNA with +10394 *Dde I* and +10397 *Alu I* sites. The cells that survived in uridine^−^ BrdU^+^ media were found to have mtDNAs with both −10394 *Dde I* and −10397 *Alu I* sites, which were identical to those of the MSCs (Fig. 3 *C*). Taken together, the cells that survived in uridine^−^ BrdU^+^ media were trans-mitochondrial hybrid 143B cells (TM143B).

### Functional characteristics of recuperated 143B

**Figure 4:**
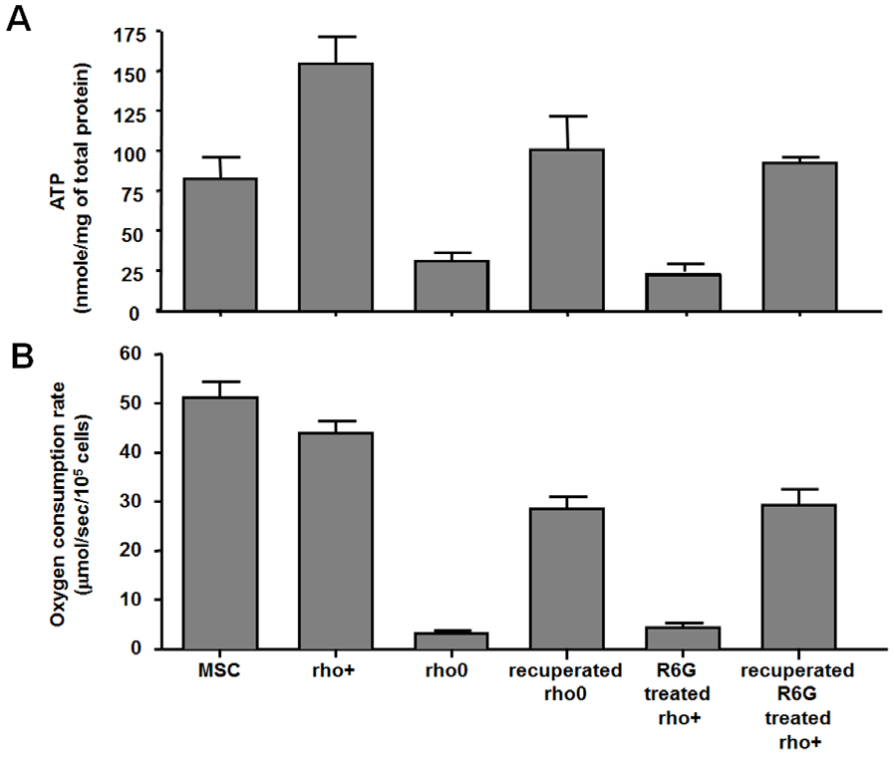
Characteristics of the recuperated TM143B cells. (A) 143B ρ^0^ cells and R6G-treated 143B ρ^+^ cells showed very low intracellular ATP content. Restored cells (TM143B) from both conditions contained much higher ATP content. (B) Oxygen consumption rates show a pattern similar to that of intracellular ATP content. Restored cells from both conditions had much higher oxygen consumption rates.

143B ρ^0^ and R6G-treated 143B ρ^+^ cells showed very low intracellular ATP content (Fig. 4 *A*). The recuperated 143B cells from ρ^0^ and R6G-treated 143B ρ^+^ cells exhibited a 3.3- and 3.7-fold increase in intracellular ATP levels, respectively (Fig. 4 *A*). The oxygen consumption rate was markedly decreased in both the 143B ρ^0^ and R6G-treated 143B ρ^+^ cells. However, the recuperated 143B cells showed an approximate 7–10-fold increase in oxygen consumption rates (Fig. 4 *B*).

### Morphology and migratory ability of MSCs

**Figure 5:**
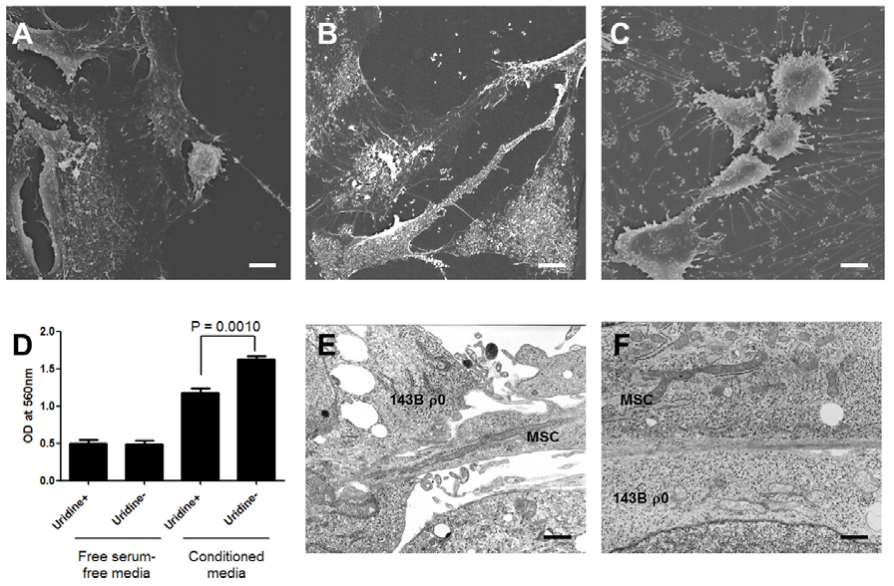
Characteristics of the MSCs in conditioned media from 143B ρ^0^ cells. A scanning electron microscopic examination showed a marked difference in the morphology of the MSCs in each conditioned medium (magnified at ×1,000; scale bars represent 10 µm). The MSCs in their own growth medium (A); after 24 h (B) and 48 h (C) in conditioned media, which were prepared by treating 143B ρ^0^ cells with uridine-free media for 48 h. The MSCs showed an increased migration in the conditioned media without uridine supplementation (D). A transmission electron microscopic examination was done after two days of Stage I coculture (magnified at ×9,000; scale bars represent 1 µm). There were intercellular contacts between ρ^0^ and the MSCs by either cytoplasmic process (E) or broad surface contact (F).

A scanning EM examination was done on MSCs cultured for 24 h in their own culture medium (DMEM) or in conditioned media, which were prepared by treating 143B ρ^0^ cells with uridine-free media for 24 or 48 h. There was a marked difference in the morphology of the MSCs in each medium: The MSCs in their own medium looked like usual fibroblasts (Fig. 5 *A*), but those in the conditioned media of 143B ρ^0^ cells looked activated and outstretched numerous cellular projections, especially when cultured in the 48 h conditioned medium (Fig. 5 *B* and *C*). Some MSCs deposited small droplets onto the tissue culture plates, which was more remarkable in the 48 h conditioned medium (Fig. 5 *C*). In a cell migration assay, MSCs showed a significantly increased migration in the conditioned media of 143B ρ^0^ cells under no uridine supplementation, compared to under uridine supplementation (1.63±0.11 vs. 1.17±0.15 in arbitrary units, n = 6, *p* = 0.031 by Wilcoxon signed rank test) (Fig. 5 *D*).

A transmission EM examination was done after three days of Stage I coculture. There were intercellular contacts between 143B ρ^0^ and MSCs by cytoplasmic processes (Fig. 5 *E*) or broad surface contact (Fig. 5 *F*); however, it is uncertain whether the mitochondria around the cellular contact point were transferred to the 143B ρ^0^ cells.

### Coculture of MSCs with cybrids with pathogenic mtDNA mutations

The cybrids harboring either A3243G mutation or 4,977 bp deletion (both characterized by −10394 *Dde I* and −10397 *Alu I*) did not show any massive cell death following the removal of uridine, even in the condition of high mutation load (e.g., homoplasmy or ∼100% of 3243G or ≥60∼80% of mtDNA with 4,977 bp deletion). Survival in the restrictive medium may be explained by some residual mitochondrial function of cybrids harboring those mutations: the relative ATP content was 20.8±7.8%, 70.7±3.7%, and 56.7±3.8% in 143B ρ^0^ cells, cybrids with ∼100% of 3243G, and cybrids with ∼80% of 4,977 bp deleted mtDNA, respectively, compared to their own wild-type controls (n = 4); the relative oxygen consumption rate was 7.5±1.4%, 28.4±3.3%, and 24.2±9.9% in 143B ρ^0^ cells, cybrids with ∼100% of 3243G, and cybrids with ∼80% of 4,977 bp deleted mtDNA, respectively, compared to their own controls (n = 4). Following the same scheme shown in Fig. 1, by using a PCR-RFLP analysis, we could not find any evidence of the transfer of mtDNA from the MSCs characterized by +10394 *Dde I* and +10397 *Alu I* restriction sites (data not shown).

### Time-course gene expression profile of the MSCs in conditioned media from 143B ρ^0^ cells

Gene expression profiles of MSCs at 0, 6, 12, 24, and 48 h after treatment with conditioned media, which were obtained by treating 143B ρ^0^ cells with uridine-free media for 48 h. We found 2,653 genes showing statistically differential expression in any of the five time points by Bayes-ANOVA (false discovery rate<0.001). Using the self-organizing map (SOM) clustering method, we grouped the 2,653 genes into 16 clusters by their expression similarity across time series (Fig. S1). To assign the biological meaning of each cluster, we performed a gene ontology (GO)-based enrichment analysis. The genes in clusters 1, 4, 6, 12, 13, 15, and 16 were found to be mainly involved in *differentiation*, *intracellular transport*, *actin-filament regulation*, *chemotaxis*, *microtubule regulation*, *synapse and cysteine metabolism*, and *response to stimuli*, respectively (Fig. S1 and Tables S3, S4, S5, S6, S7, S8, S9).

To visualize the global relationship of individual clusters, we performed a BioLattice [13] analysis with 49 clusters created by SOM (Fig. S2), which was reanalyzed by using a more detailed 7×7 grid format with the same dataset used in Fig. S1. BioLattice enables a better biological interpretation of microarray gene expression data by organizing clusters into a unified lattice of concepts [13]. Twelve clusters having at least one significant GO term annotated in the biological process category (*p*<0.001) were organized with 15 concepts created into a concept lattice (Fig. S3, Tables S10 and S11). A core-periphery analysis determined “core” concepts of the MSCs to the response to the stimuli of ρ^0^-conditioned media such as C2, C3, and C13 (marked in red in Fig. S3), which would to be more biologically relevant than the “periphery” concepts. More comprehensive analysis results for other GO categories at various threshold levels are available at our web site (http://www.snubi.org/publication/msc).

## Discussion

By using biochemical selection methods, we hereby showed that MSCs can transfer mitochondria to the cells lacking mtDNA, which is consistent with the previous report [2]. All recuperated cells were TM143B cybrid cells that contained the nuclear genome of 143B cells and mtDNAs from the MSCs. Also, we demonstrated the same phenomenon can take place in a condition with severe mitochondrial dysfunction caused by non-genetic mitochondrial toxin (R6G). However, such phenomena were not observed in the cells harboring pathogenic mtDNA mutations such as A3243G mutation or 4,977 bp deletion. These observations suggest that the mitochondrial transfer may occur only in a situation with virtually absent mitochondrial function. However, we could not exclude the possibility that a very small number of mtDNAs from MSCs, which might be far below the detection limit, might be transferred to those cybrid cells harboring A3243G mutation or 4,977 bp deletion. Since it was proposed that mitochondrial diseases caused by mtDNA mutations could be treated by a process of inter-mitochondrial complementation through transferring normal mitochondria [14], [15], it is imperative to find a way to facilitate the mitochondrial transfer to the cells with mitochondrial dysfunction in humans. In this regard, it is very intriguing that partial cell fusion and mitochondrial transfer may occur between human multipotent stem cells and fully differentiated murine cardiomyocytes, rendering the cardiomyocytes reprogrammed toward a progenitor-like state [16].

Although a restoration of mitochondrial function in the cells with severely compromised mitochondrial function was reported [2], it remains uncertain whether the cells were rescued by the transfer of intact mitochondrial particles or by the transfer of mtDNA alone. We addressed this question with an experiment using R6G as a mitochondrial toxin. R6G is a fluorescent dye that binds tightly to the inner mitochondrial membrane and therefore is a potent inhibitor of oxidative phosphorylation [4]. It has been used to remove the mitochondrial influence from one parental cell line prior to the construction of cybrids using hamster [7], mouse [5], and human cells [6]. Interestingly, in R6G-treated cells, mtDNA was still detected after short-term exposure [6]. Consistent with this report, we observed the presence of mtDNA in the R6G-treated cells by PCR in this study. Since the rescued cells after R6G treatment were revealed to contain mtDNAs from the MSCs, the mtDNAs of the 143B ρ^+^ cells might have been eventually removed following irreversible mitochondrial damage. Therefore, our results suggest that the mitochondrial function was restored by the transfer of intact mitochondrial particles rather than the transfer of mtDNA alone.

There is growing body of evidence on the cell fusion. Adult and embryonic stem cells have been reported to fuse with cardiomyocytes, hepatocytes, and neurons [17], [18], [19], [20], which might contribute to the differentiation, plasticity, or maintenance of these cell types. Permanent cell fusion may result in cells with unique karyotypes (e.g., binucleated heterokaryons or mononucleated hyperploid synkaryons) [21], while partial cell fusion allows transient direct intercellular communication [22], [23] and exchange or transfer of multiple protein complexes [24] and even subcellular organelles [25]. Since the recuperated cells have exactly the same fingerprint pattern of the nuclear DNA of 143B cells, the nuclear fusion between 143B-derived cells and MSCs was unlikely to take place. Instead, we found that the sequences of mtDNAs of the recuperated cells were identical to those of the MSCs. Therefore, we can conclude that partial cell fusion mediated the rescue of 143B-derived cells with severely compromised mitochondrial function.

The mechanism of mitochondrial transfer is considered as an active cellular process, since no restoration of mitochondrial function in cells lacking mtDNA was observed when cultured with isolated mitochondria or platelets (platelets have mitochondria but lack nuclei) [2]. This suggests that the response of the MSCs to the stimuli from ρ^0^ cells in stress would be crucial in the mitochondrial transfer. In this regard, our microarray data might shed light on perspectives explaining possible mechanisms. The differentially expressed genes—along with the time-series following the treatment of conditioned media obtained by treating 143B ρ^0^ cells with uridine-free media for 48 h—were largely examined within the context of chemotaxis, actin-filament regulation, microtubule regulation, and cellular responses to various stimuli. Indeed, it was recently revealed that f-actin and microtubule filaments are essential in the process of partial cell fusion and subsequent mitochondria transfer [16]. Also, we could not find any rapidly growing colonies in the cell fusion experiment with cytochalasin B, an inhibitor of chemotaxis and cytoskeletal assembly. Taken together, some unknown factors secreted from the cells with virtually absent mitochondrial function might activate the MSC, prompting mitochondrial transfer by active cellular processes.

Spees *et al.* [2] suggested that MSCs might transfer mitochondria by directing cytoplasm toward ρ^0^ cells, either as cytoplasmic extensions or broad areas of cellular contact. Consistent with these findings, we also found in a transmission EM examination that many MSCs were adjacent to ρ^0^ cells with cytoplasmic extensions that contained elongated mitochondria or broad areas of plasma membrane contact. However, the snap shot image cannot tell whether mitochondria were transferred to ρ^0^ cells by these processes. Recently, in a study adopting human multipotent adipose-derived stem cells expressing Cre recombinase and murine cardiomyocytes expressing LacZ gene under the control of an internal stop codon flanked by loxP sites, partial cell fusion and mitochondrial transfer through a very thin tube-like structure were demonstrated by Xgal staining and time-lapse video microscopy tracking MitoTracker-stained mitochondria [16]. All together, either cytoplasmic extensions or broad cell-to-cell contact may serve as the route of the intercellular mitochondrial transfer.

Although there are several theoretical strategies to treat mitochondriopathies due to mtDNA mutations by gene therapy [26], the practical implementation of such strategies is largely hampered by the unique characteristics of mtDNA (polyplasmy and heteroplasmy) [27]. Indeed, as of yet, nobody succeeded to deliver DNA into mitochondria in a heritable manner [27]. However, it has been suggested that mitochondrial diseases elicited by mtDNA mutations could be treated by exploiting the inter-mitochondrial complementation through exchange of genetic materials between mitochondria with normal and mutant mtDNAs [14], [15] In this regard, the elucidation of the mechanism of the mitochondrial transfer by human MSCs will provide a potential cell therapy-based mitochondrial gene therapy for human mtDNA diseases.

Although it is yet to be elucidated what signal is involved in the mitochondrial transfer, we found (i) MSCs restores mitochondrial function of mtDNA-less cells and mitochondrial toxin-treated cells, (ii) the mitochondrial transfer process may be a part of partial cell fusion which is not accompanied by nuclear fusion, (iii) the restoration of mitochondrial function may be mediated by the transfer of intact mitochondrial particles not by the transfer of mtDNA alone, (iv) the mitochondrial transfer to the cells harboring pathogenic mtDNA mutations may not take place in our experimental condition, (v) The process of mitochondrial transfer may be mediated by active cellular mechanism(s) including chemotaxis and other cellular responses to various stimuli. Further studies are needed to reveal the molecular mechanisms of mitochondrial transfer.

## Supporting Information

Figure S1 **Patterns for differentially expressed genes in a time-dependent manner.** The time-differential 2,653 genes were grouped into 16 clusters using SOM. The 16 clusters were arranged into a 4×4 rectangular grid so that neighboring cells had similar expression. The line graph in each cell represents the relative expression of all genes in the corresponding cluster with mean ± standard error; a transverse line denotes the mean value of expression levels of all genes in the cluster.
(PDF)

Figure S2 **Patterns for differentially expressed genes in a time-dependent manner.** The time-differential 2,653 genes are clustered into 49 groups using Self-Organizing Maps technique. The 49 clusters are arranged into a 7×7 rectangular grid so that neighboring cells have similar expression. Cluster 302 is missing, since no genes were allocated in this category. The line graph in each cell represents the average expression of all genes in the corresponding cluster with mean ± standard error.
(PDF)

Figure S3 **BioLattice analysis.** Concept lattice was constructed from the 49 clusters shown in Figure S2 with significant GO annotations from the MSCs gene-expression dataset. Only 12 among the 49 clusters demonstrate at least one significant GO term(s) (P<0.001) in the biological process category. Overall, the dataset shows 56 significant annotations with 56 unique GO terms. The core–periphery substructures are marked with colors (i.e., core in red, communicating in green, peripheral in gray and independent in yellow). The numbers in the lower semicircles is the cluster numbers shown in Figure S2. The numbers of the upper semicircles are the concept ID made by the current Biolattice analysis.
(PDF)

Table S1 DNA fingerprinting analysis.
(DOC)

Table S2 mtDNA D-loop sequence.
(DOC)

Table S3 GO annotations with P-value<0.0001 in C1 of 4×4 clusters by SOM clustering.
(DOC)

Table S4 GO annotations with P-value<0.0001 in C4 of 4×4 clusters by SOM clustering.
(DOC)

Table S5 GO annotations with P-value<0.0001 in C6 of 4×4 clusters by SOM clustering.
(DOC)

Table S6 GO annotations with P-value<0.0001 in C12 of 4×4 clusters by SOM clustering.
(DOC)

Table S7 GO annotations with P-value<0.0001 in C13 of 4×4 clusters by SOM clustering.
(DOC)

Table S8 GO annotations with P-value<0.0001 in C15 of 4×4 clusters by SOM clustering.
(DOC)

Table S9 GO annotations with P-value<0.0001 in C16 of 4×4 clusters by SOM clustering.
(DOC)

Table S10 Prominent sub-lattice analysis of a concept lattice shown in Fig. S3.
(DOC)

Table S11 Core-periphery analysis of a concept lattice shown in Fig. S3.
(DOC)
