# Supplementary figures and images for: Mesenchymal Stem Cells Transfer Mitochondria to the Cells with Virtually No Mitochondrial Function but Not with Pathogenic mtDNA Mutations
Source: PLoS One. 2012 Mar 6;7(3):e32778. doi: 10.1371/journal.pone.0032778 (PMC3295770; doi:10.1371/journal.pone.0032778)

Self-organizing map (SOM) clustering

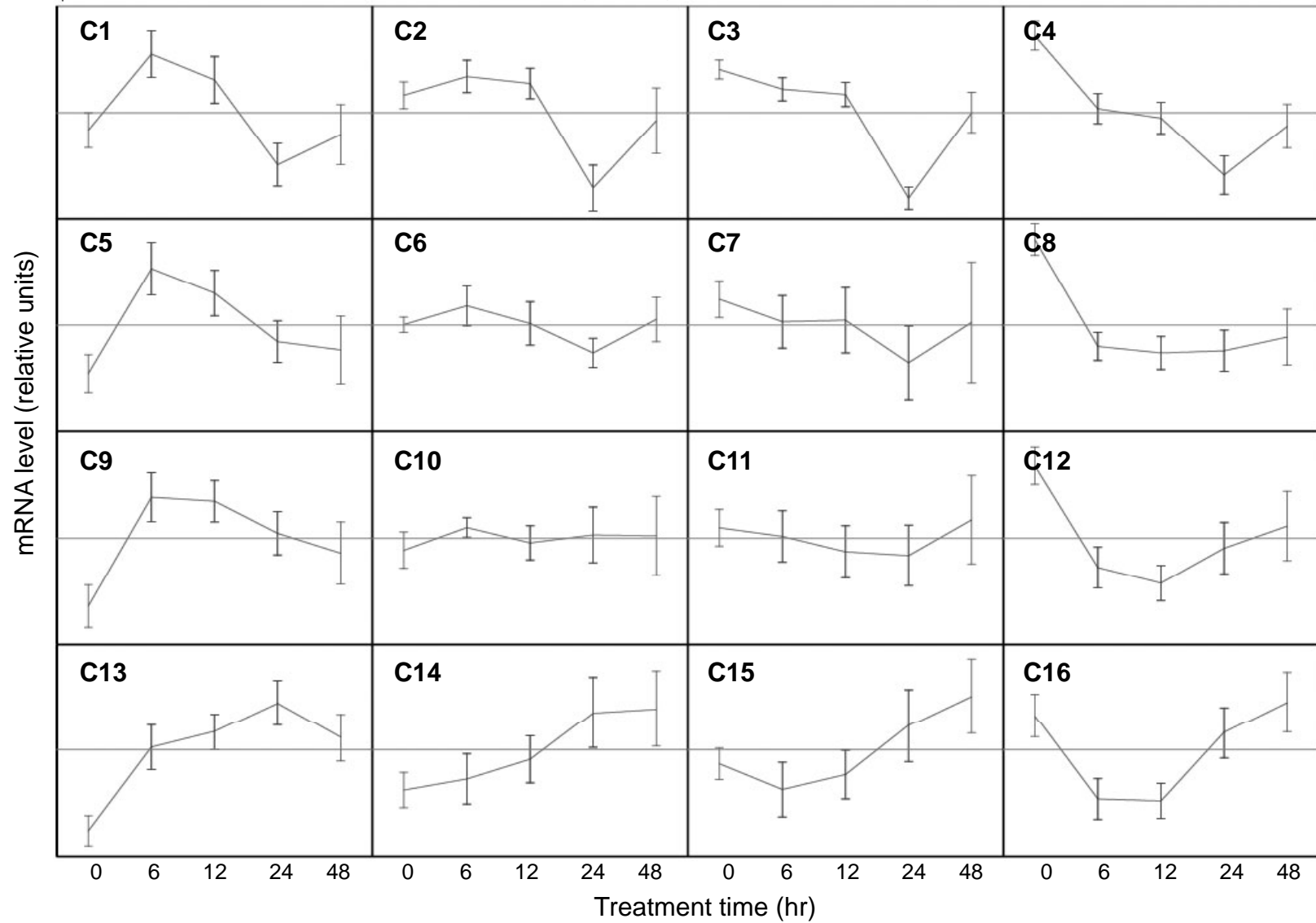

Supplement: Figure S1 — Patterns for differentially expressed genes in a time-dependent manner. The time-differential 2,653 genes were grouped into 16 clusters using SOM. The 16 clusters were arranged into a 4×4 rectangular grid so that neighboring cells had similar expression. The line graph in each cell represents the relative expression of all genes in the corresponding cluster with mean ± standard error; a transverse line denotes the mean value of expression levels of all genes in the cluster. (PDF) [file pone.0032778.s001.pdf]

# Self-organizing map (SOM) clustering

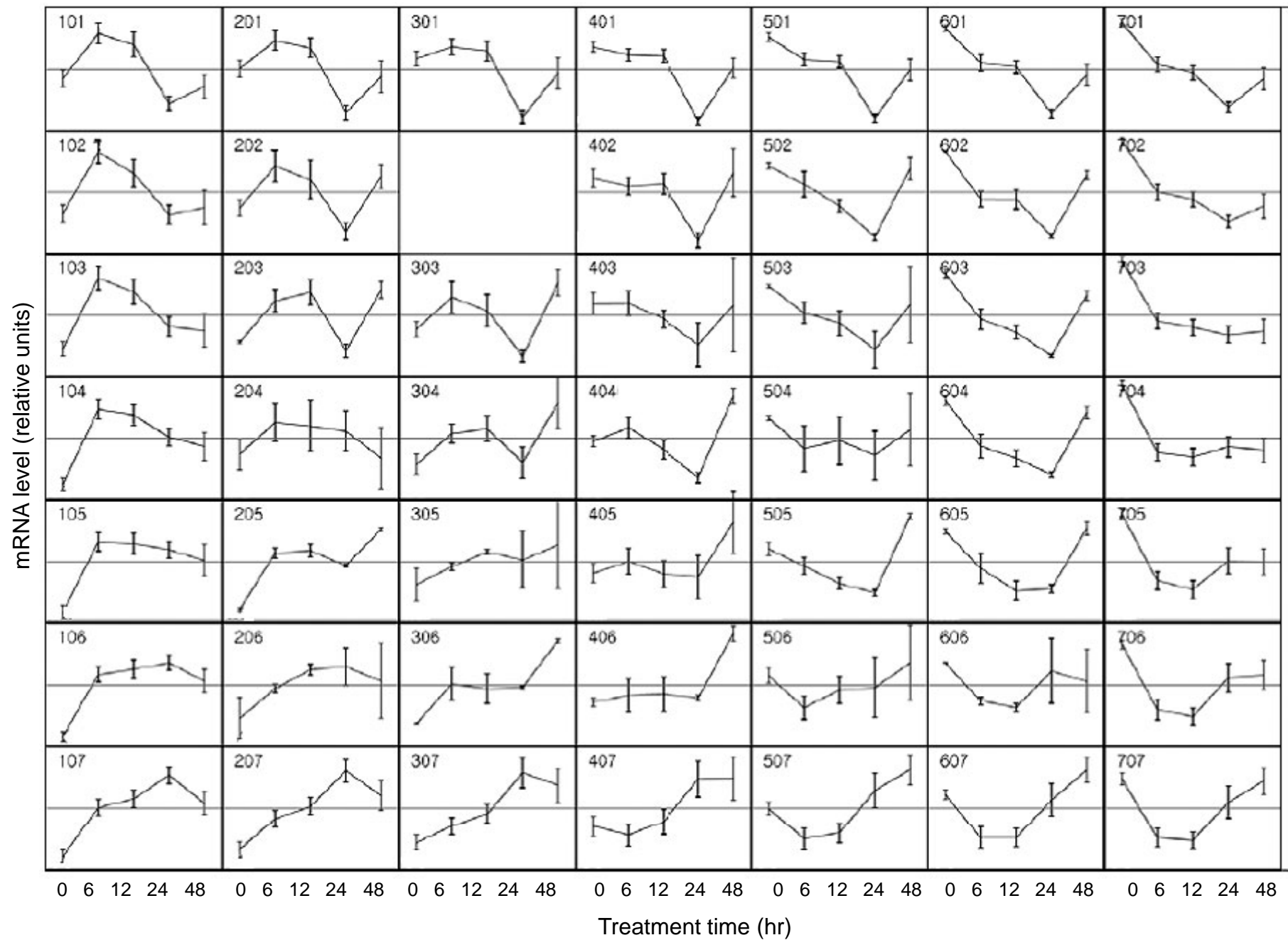

Supplement: Figure S2 — Patterns for differentially expressed genes in a time-dependent manner. The time-differential 2,653 genes are clustered into 49 groups using Self-Organizing Maps technique. The 49 clusters are arranged into a 7×7 rectangular grid so that neighboring cells have similar expression. Cluster 302 is missing, since no genes were allocated in this category. The line graph in each cell represents the average expression of all genes in the corresponding cluster with mean ± standard error. (PDF) [file pone.0032778.s002.pdf]

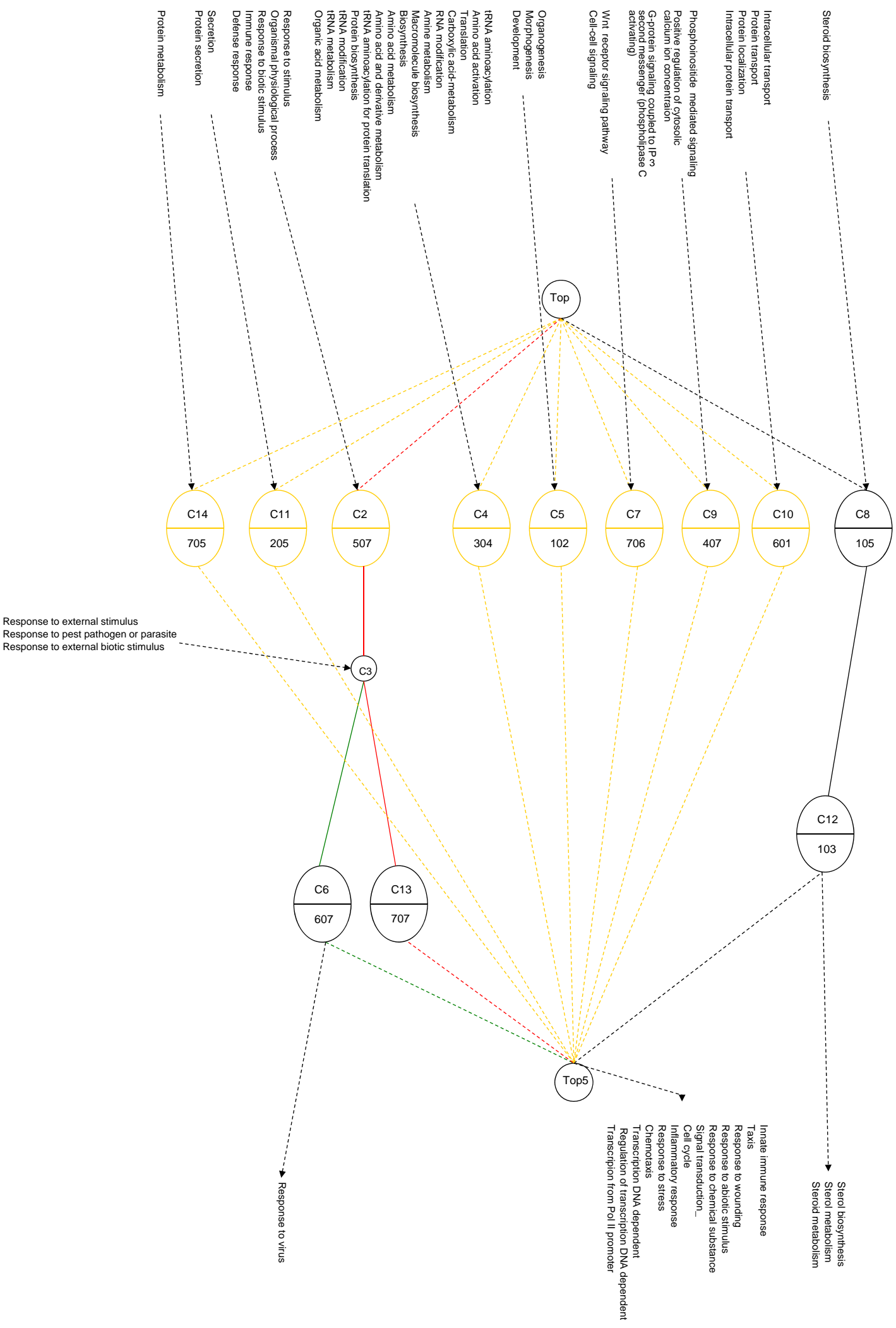

Supplement: Figure S3 — BioLattice analysis. Concept lattice was constructed from the 49 clusters shown in Figure S2 with significant GO annotations from the MSCs gene-expression dataset. Only 12 among the 49 clusters demonstrate at least one significant GO term(s) (P<0.001) in the biological process category. Overall, the dataset shows 56 significant annotations with 56 unique GO terms. The core–periphery substructures are marked with colors (i.e., core in red, communicating in green, peripheral in gray and independent in yellow). The numbers in the lower semicircles is the cluster numbers shown in Figure S2. The numbers of the upper semicircles are the concept ID made by the current Biolattice analysis. (PDF) [file pone.0032778.s003.pdf]
