# Supplementary material for: Mesenchymal Stem Cells Transfer Mitochondria to the Cells with Virtually No Mitochondrial Function but Not with Pathogenic mtDNA Mutations
Source: PLoS One. 2012 Mar 6;7(3):e32778. doi: 10.1371/journal.pone.0032778 (PMC3295770; doi:10.1371/journal.pone.0032778)
Supplement: Table S1 — DNA fingerprinting analysis. (DOC) [file pone.0032778.s004.doc]

Table S1. DNA fingerprinting analysis

| Markers | MSC* | 143B ρ^0^ | Recuperated† |
| --- | --- | --- | --- |
| D1S80 | 33, 18 | 24, 18 | 24, 18 |
| D3S1744 | 16, 16 | 17, 17 | 17, 17 |
| D3S2406 | 34, 31 | 33, 33 | 33, 33 |
| D4S2368 | 12, 11 | 11, 11 | 11, 11 |
| D5S818 | 13, 12 | 13, 13 | 13, 13 |
| D6S1043 | 13, 11 | 18, 18 | 18, 18 |
| D7S821 | 16, 14 | 17, 12 | 17, 12 |
| D9S925 | 16, 14 | 11, 11 | 11, 11 |
| D8S1179 | 10, 14 | 14, 14 | 14, 14 |
| D12S391 | 19, 18 | 21, 20 | 21, 20 |
| D13S317 | 08, 08 | 12, 12 | 12, 12 |
| D17S5 | 03, 01 | 01, 01 | 01, 01 |
| D18S51 | 13, 15 | 17, 17 | 17, 17 |
| D19S253 | 13, 07 | 12, 12 | 12, 12 |
| D21S11 | 30, 32.2 | 31.2, 32.2 | 31.2, 32.2 |
| TC-11 | 09, 09 | 06, 06 | 06, 06 |
| TPOX | 11, 08 | 11, 11 | 11, 11 |
| CSF1P0 | 11, 11 | 12, 12 | 12, 12 |
| vWA | 14, 17 | 18, 18 | 18, 18 |
| ATA28C05 | 11 | 11, 11 | 11, 11 |
| FGA | 21, 24 | 24, 24 | 24, 24 |
| DXS6797 | 20 | 20, 20 | 20, 20 |
| DXS6804 | 13 | 13, 13 | 13, 13 |
| DXS7133 | 09 | 09, 09 | 09, 09 |
| DXS9898 | 10 | 11, 11 | 11, 11 |
| GATA144D04 | 11 | 07, 07 | 07, 07 |

^*^MSC, mesenchymal stem cell; ^†^Recuperated, cells survived after Stage I and II coculture procedures
