# Supplementary material for: Mesenchymal Stem Cells Transfer Mitochondria to the Cells with Virtually No Mitochondrial Function but Not with Pathogenic mtDNA Mutations
Source: PLoS One. 2012 Mar 6;7(3):e32778. doi: 10.1371/journal.pone.0032778 (PMC3295770; doi:10.1371/journal.pone.0032778)
Supplement: Table S2 — mtDNA D-loop sequence. (DOC) [file pone.0032778.s005.doc]

Table S2. mtDNA D-loop sequence

| mtDNA nucleotide position | Cambridge reference sequence | MSC* | 143B ρ^0^ | Recuperated† |
| --- | --- | --- | --- | --- |
| 15773 | G | A | - | A |
| 16189 | T | C | - | C |
| 16223 | C | T | - | T |
| 16257 | C | A | - | A |
| 16261 | C | T | - | T |
| 16292 | C | T | - | T |
| 73 | A | G | - | G |
| 150 | C | T | - | T |
| 263 | A | G | - | G |
| 750 | A | G | - | G |
| 801 | A | G | - | G |
| 309-310 | - | CC | - | CC |
| 315-316 | - | C | - | C |

^*^MSC, mesenchymal stem cell; ^†^Recuperated, cells survived after Stage I and II coculture procedures
