# Supplementary material for: Mesenchymal Stem Cells Transfer Mitochondria to the Cells with Virtually No Mitochondrial Function but Not with Pathogenic mtDNA Mutations
Source: PLoS One. 2012 Mar 6;7(3):e32778. doi: 10.1371/journal.pone.0032778 (PMC3295770; doi:10.1371/journal.pone.0032778)
Supplement: Table S3 — GO annotations with P-value<0.0001 in C1 of 4×4 clusters by SOM clustering. (DOC) [file pone.0032778.s006.doc]

Table S3. GO annotations with P-value < 0.0001 in C1 of 4 × 4 clusters by SOM clustering

| Name | Frequency | P value |
| --- | --- | --- |
| Organogenesis | 25% | 1.56 × 10^-11^ |
| Organ development | 25% | 1.56 × 10^-11^ |
| Morphogenesis | 27% | 5.40 × 10^-11^ |
| Development | 31% | 2.78 × 10^-8^ |
| Regulation of hormone secretion | 3% | 4.80 × 10^-7^ |
| Muscle development | 8% | 1.42 × 10^-6^ |
| Hormone secretion | 3% | 5.20 × 10^-6^ |
| Regulation of follicle-stimulating hormone secretion | 2% | 5.61 × 10^-6^ |
| Negative regulation of follicle-stimulating hormone secretion | 2% | 5.61 × 10^-6^ |
| Follicle-stimulating hormone secretion | 2% | 5.61 × 10^-6^ |
| Negative regulation of hormone secretion | 2% | 8.00 × 10^-6^ |
| Neurogenesis | 11% | 9.10 × 10^-6^ |
| Negative regulation of secretion | 2% | 1.46 × 10^-5^ |
| Regulation of secretion | 3% | 1.63 × 10^-5^ |
| Extracellular transport | 1% | 1.68 × 10^-5^ |
| Blood coagulation | 5% | 2.09 × 10^-5^ |
| Coagulation | 5% | 2.52 × 10^-5^ |
| Wound healing | 5% | 3.02 × 10^-5^ |
| Hemostasis | 5% | 3.51 × 10^-5^ |
| Regulation of body fluids | 5% | 6.43 × 10^-5^ |
