# Supplementary material for: Mesenchymal Stem Cells Transfer Mitochondria to the Cells with Virtually No Mitochondrial Function but Not with Pathogenic mtDNA Mutations
Source: PLoS One. 2012 Mar 6;7(3):e32778. doi: 10.1371/journal.pone.0032778 (PMC3295770; doi:10.1371/journal.pone.0032778)
Supplement: Table S4 — GO annotations with P-value<0.0001 in C4 of 4×4 clusters by SOM clustering. (DOC) [file pone.0032778.s007.doc]

Table S4. GO annotations with P-value < 0.0001 in C4 of 4 × 4 clusters by SOM clustering

| Name | Frequency | P value |
| --- | --- | --- |
| Intracellular transport | 12% | 4.95 × 10^-11^ |
| Nuclear transport | 5% | 4.62 × 10^-9^ |
| Nucleocytoplasmic transport | 5% | 1.34 × 10^-8^ |
| Protein targeting | 5% | 5.71 × 10^-8^ |
| Intracellular protein transport | 8% | 2.78 × 10^-7^ |
| Nuclear export | 3% | 2.80 × 10^-7^ |
| Protein transport | 10% | 6.39 × 10^-7^ |
| Establishment of protein localization | 10% | 6.74 × 10^-7^ |
| RNA metabolism | 8% | 1.31 × 10^-6^ |
| Protein localization | 10% | 1.41 × 10^-6^ |
| Regulation of protein-nucleus export | 1% | 6.03 × 10^-6^ |
| Regulation of nucleocytoplasmic transport | 2% | 8.34 × 10^-6^ |
| mRNA metabolism | 5% | 1.12 × 10^-5^ |
| RNA processing | 7% | 1.38 × 10^-5^ |
| Peptidyl-serine phosphorylation | 1% | 1.49 × 10^-5^ |
| Peptidyl-serine modification | 1% | 1.49 × 10^-5^ |
| Regulation of protein transport | 1% | 1.87 × 10^-5^ |
