# Supplementary material for: Mesenchymal Stem Cells Transfer Mitochondria to the Cells with Virtually No Mitochondrial Function but Not with Pathogenic mtDNA Mutations
Source: PLoS One. 2012 Mar 6;7(3):e32778. doi: 10.1371/journal.pone.0032778 (PMC3295770; doi:10.1371/journal.pone.0032778)
Supplement: Table S5 — GO annotations with P-value<0.0001 in C6 of 4×4 clusters by SOM clustering. (DOC) [file pone.0032778.s008.doc]

Table S5. GO annotations with P-value < 0.0001 in C6 of 4 × 4 clusters by SOM clustering

| Name | Frequency | P value |
| --- | --- | --- |
| Negative regulation of actin filament polymerization | 50% | 5.79 × 10^-5^ |
| Sequestering of actin monomers | 50% | 5.79 × 10^-5^ |
