# Supplementary material for: Mesenchymal Stem Cells Transfer Mitochondria to the Cells with Virtually No Mitochondrial Function but Not with Pathogenic mtDNA Mutations
Source: PLoS One. 2012 Mar 6;7(3):e32778. doi: 10.1371/journal.pone.0032778 (PMC3295770; doi:10.1371/journal.pone.0032778)
Supplement: Table S6 — GO annotations with P-value<0.0001 in C12 of 4×4 clusters by SOM clustering. (DOC) [file pone.0032778.s009.doc]

Table S6. GO annotations with P-value < 0.0001 in C12 of 4 × 4 clusters by SOM clustering

| Name | Frequency | P value |
| --- | --- | --- |
| Positive chemotaxis | 3% | 2.40 × 10^-6^ |
| Regulation of positive chemotaxis | 3% | 2.40 × 10^-6^ |
| Positive regulation of positive chemotaxis | 3% | 2.40 × 10^-6^ |
| Induction of positive chemotaxis | 3% | 2.40 × 10^-6^ |
| Regulation of chemotaxis | 3% | 4.69 × 10^-6^ |
| Positive regulation of chemotaxis | 3% | 4.69 × 10^-6^ |
| Regulation of cellular process | 41% | 1.52 × 10^-5^ |
| Regulation of vascular endothelial growth factor receptor signaling pathway | 2% | 5.68 × 10^-5^ |
| Positive regulation of vascular endothelial growth factor receptor signaling pathway | 2% | 5.68 × 10^-5^ |
| Vascular endothelial growth factor receptor signaling pathway | 2% | 5.68 × 10^-5^ |
| Regulation of cellular physiological process | 38% | 5.88 × 10^-5^ |
| Regulation of biological process | 41% | 9.49 × 10^-5^ |
