# Supplementary material for: Mesenchymal Stem Cells Transfer Mitochondria to the Cells with Virtually No Mitochondrial Function but Not with Pathogenic mtDNA Mutations
Source: PLoS One. 2012 Mar 6;7(3):e32778. doi: 10.1371/journal.pone.0032778 (PMC3295770; doi:10.1371/journal.pone.0032778)
Supplement: Table S7 — GO annotations with P-value<0.0001 in C13 of 4×4 clusters by SOM clustering. (DOC) [file pone.0032778.s010.doc]

Table S7. GO annotations with P-value < 0.0001 in C13 of 4 × 4 clusters by SOM clustering

| Name | Frequency | P value |
| --- | --- | --- |
| Cell organization and biogenesis | 13% | 1.57 × 10^-6^ |
| Negative regulation of microtubule depolymerization | 1% | 1.03 × 10^-5^ |
| Regulation of microtubule polymerization or depolymerization | 1% | 1.03 × 10^-5^ |
| Negative regulation of microtubule polymerization or depolymerization | 1% | 1.03 × 10^-5^ |
| Regulation of microtubule depolymerization | 1% | 1.03 × 10^-5^ |
| Negative regulation of microtubule depolymerization | 1% | 1.03 × 10^-5^ |
| Protein depolymerization | 1% | 1.09 × 10^-5^ |
| Negative regulation of cell organization and biogenesis | 1% | 1.47 × 10^-5^ |
| Cytoskeleton organization and biogenesis | 6% | 1.67 × 10^-5^ |
| Microtubule depolymerization | 1% | 1.95 × 10^-5^ |
| Microtubule polymerization or depolymerization | 1% | 1.95 × 10^-5^ |
| Organelle organization and biogenesis | 11% | 2.29 × 10^-5^ |
