# Supplementary material for: Mesenchymal Stem Cells Transfer Mitochondria to the Cells with Virtually No Mitochondrial Function but Not with Pathogenic mtDNA Mutations
Source: PLoS One. 2012 Mar 6;7(3):e32778. doi: 10.1371/journal.pone.0032778 (PMC3295770; doi:10.1371/journal.pone.0032778)
Supplement: Table S8 — GO annotations with P-value<0.0001 in C15 of 4×4 clusters by SOM clustering. (DOC) [file pone.0032778.s011.doc]

Table S8. GO annotations with P-value < 0.0001 in C15 of 4 × 4 clusters by SOM clustering

| Name | Frequency | P value |
| --- | --- | --- |
| Synaptic transmission, cholinergic | 5% | 7.88 × 10^-6^ |
| Cysteine biosynthesis from serine | 3% | 1.01 × 10^-5^ |
| Cysteine biosynthesis via cystathione | 3% | 1.01 × 10^-5^ |
| Protein amino acid ADP-ribosylation | 6% | 1.12 × 10^-5^ |
| Nerve-nerve synaptic transmission | 5% | 2.37 × 10^-5^ |
| Amino acid biosynthesis | 6% | 5.93 × 10^-5^ |
| Cysteine biosynthesis | 3% | 7.05 × 10^-5^ |
| Serine family amino acid biosynthesis | 5% | 7.10 × 10^-5^ |
| L-serine metabolism | 5% | 8.70 × 10^-5^ |
