# Supplementary material for: Mesenchymal Stem Cells Transfer Mitochondria to the Cells with Virtually No Mitochondrial Function but Not with Pathogenic mtDNA Mutations
Source: PLoS One. 2012 Mar 6;7(3):e32778. doi: 10.1371/journal.pone.0032778 (PMC3295770; doi:10.1371/journal.pone.0032778)
Supplement: Table S9 — GO annotations with P-value<0.0001 in C16 of 4×4 clusters by SOM clustering. (DOC) [file pone.0032778.s012.doc]

Table S9. GO annotations with P-value < 0.0001 in C16 of 4 × 4 clusters by SOM clustering

| Name | Frequency | P value |
| --- | --- | --- |
| Response to stimulus | 32% | 1.13 × 10^-21^ |
| Response to biotic stimulus | 22% | 1.05 × 10^-20^ |
| Immune response | 19% | 3.13 × 10^-20^ |
| Defense response | 19% | 9.09 × 10^-19^ |
| Response to stress | 21% | 2.50 × 10^-17^ |
| Organismal physiological process | 27% | 2.47 × 10^-14^ |
| Response to pest, pathogen or parasite | 12% | 6.44 × 10^-14^ |
| Response to external stimulus | 20% | 9.85 × 10^-14^ |
| Response to external biotic stimulus | 12% | 1.92 × 10^-13^ |
| Response to wounding | 10% | 6.84 × 10^-11^ |
| Negative regulation of physiological process | 12% | 5.34 × 10^-9^ |
| Regulation of cell cycle | 11% | 5.49 × 10^-9^ |
| Negative regulation of biological process | 13% | 2.04 × 10^-8^ |
| Response to virus | 3% | 4.09 × 10^-8^ |
| Negative regulation of cellular physiological process | 11% | 7.18 × 10^-8^ |
| Inflammatory response | 5% | 2.89 × 10^-7^ |
| Negative regulation of cellular process | 11% | 3.40 × 10^-7^ |
| Cell proliferation | 10% | 8.42 × 10^-7^ |
| Regulation of physiological process | 34% | 8.70 × 10^-7^ |
| Coagulation | 4% | 2.17 × 10^-6^ |
| Response to chemical substance | 5% | 2.32 × 10^-6^ |
| Regulation of biological process | 36% | 3.33 × 10^-6^ |
| Chemotaxis | 4% | 3.91 × 10^-6^ |
| Taxis | 4% | 3.91 × 10^-6^ |
| Regulation of cellular physiological process | 33% | 4.27 × 10^-6^ |
| Cell cycle | 12% | 4.53 × 10^-6^ |
| Regulation of cellular process | 34% | 7.06 × 10^-6^ |
| Regulation of cell proliferation | 6% | 8.89 × 10^-6^ |
| Blood coagulation | 3% | 1.12 × 10^-5^ |
| Tryptophan catabolism | 1% | 1.26 × 10^-5^ |
| Indole derivative catabolism | 1% | 1.26 × 10^-5^ |
| Indolalkylamine catabolism | 1% | 1.26 × 10^-5^ |
| Wound healing | 3% | 1.86 × 10^-5^ |
| Hemostasis | 3% | 2.27 × 10^-5^ |
| Cell-cell signaling | 8% | 2.98 × 10^-5^ |
| Prostanoid metabolism | 2% | 3.46 × 10^-5^ |
| Prostaglandin metabolism | 2% | 3.46 × 10^-5^ |
| Protein kinase cascade | 6% | 4.61 × 10^-5^ |
| I-kappab kinase/NF-kappab cascade | 4% | 5.10 × 10^-5^ |
| Regulation of body fluids | 3% | 5.12 × 10^-5^ |
| Positive chemotaxis | 1% | 5.19 × 10^-5^ |
| Regulation of positive chemotaxis | 1% | 5.19 × 10^-5^ |
| Positive regulation of positive chemotaxis | 1% | 5.19 × 10^-5^ |
| Induction of positive chemotaxis | 1% | 5.19 × 10^-5^ |
| STAT protein nuclear translocation | 1% | 7.37 × 10^-5^ |
| DNA catabolism, exonucleolytic | 1% | 7.47 × 10^-5^ |
| Cyclooxygenase pathway | 1% | 7.47 × 10^-5^ |
| Regulation of lipoprotein lipase activity | 1% | 7.47 × 10^-5^ |
| Negative regulation of lipoprotein lipase activity | 1% | 7.47 × 10^-5^ |
| Negative regulation of hydrolase activity | 1% | 7.47 × 10^-5^ |
| Negative regulation of cell proliferation | 4% | 9.22 × 10^-5^ |
