# Supplementary material for: Mesenchymal Stem Cells Transfer Mitochondria to the Cells with Virtually No Mitochondrial Function but Not with Pathogenic mtDNA Mutations
Source: PLoS One. 2012 Mar 6;7(3):e32778. doi: 10.1371/journal.pone.0032778 (PMC3295770; doi:10.1371/journal.pone.0032778)
Supplement: Table S10 — Prominent sub-lattice analysis of a concept lattice shown in Fig. S3. (DOC) [file pone.0032778.s013.doc]

Table S10. Prominent sub-lattice analysis of a concept lattice shown in Fig. S3

| The largest prominent sub-lattice has 3 nodes having clusters 507, 707 |
| --- |
| Cell cycle  Chemotaxis  Defense response  Immune response  Inflammatory response  Innate immune response  Organismal physiological process  Regulation of transcription DNA-dependent  Response to abiotic stimulus  Response to biotic stimulus  Response to chemical substance  Response to external biotic stimulus  Response to external stimulus  Response to pest pathogen or parasite  Response to stimulus  Response to stress  Response to wounding  Signal transduction  Taxis  Transcription DNA-dependent  Transcription from Pol II promoter |
| The second largest sub-lattice has 3 nodes having clusters 507, 607 |
| Defense response  Immune response  Organismal physiological_process  Response to biotic stimulus  Response to external biotic stimulus  Response to external stimulus  Response to pest pathogen or parasite  Response to stimulus  Response to virus |
| The third largest sub-lattice has 2 nodes having clusters 103, 105 |
| Steroid biosynthesis  Steroid metabolism  Sterol biosynthesis  Sterol metabolism |
| The fourth sub-lattice has 1 node having cluster 705 |
| Protein metabolism |
| The fifth sub-lattice has 1 node having cluster 205 |
| Protein secretion  Secretion |
