# Supplementary material for: Mesenchymal Stem Cells Transfer Mitochondria to the Cells with Virtually No Mitochondrial Function but Not with Pathogenic mtDNA Mutations
Source: PLoS One. 2012 Mar 6;7(3):e32778. doi: 10.1371/journal.pone.0032778 (PMC3295770; doi:10.1371/journal.pone.0032778)
Supplement: Table S11 — Core-periphery analysis of a concept lattice shown in Fig. S3. (DOC) [file pone.0032778.s014.doc]

Table S11. Core-periphery analysis of a concept lattice shown in Fig. S3

| Sub-structure | Significant annotation |
| --- | --- |
| Core | Clusters 507, 607 and 707  Cell cycle  Chemotaxis  Defense response  Immune response  Inflammatory response  Innate immune response  Organismal physiological process  Regulation of transcription dna-dependent  Response to abiotic stimulus  Response to biotic stimulus  Response to chemical substance  Response to external biotic stimulus  Response to external stimulus  Response to pest pathogen or parasite  Response to stimulus  Response to stress  Response to virus  Response to wounding  Signal transduction  Taxis  Transcription dna-dependent  Transcription from pol ii promoter |
| Communicating | Cluster |
| Peripheral | Clusters 103, 105  Steroid biosynthesis  Steroid metabolism  Sterol biosynthesis  Sterol metabolism |
| Independent | Clusters 102, 205, 304, 407, 601, 705 and 706  RNA modification  Wnt receptor signaling pathway  Amine metabolism  Amino acid activation  Amino acid and derivative metabolism  Amino acid metabolism  Biosynthesis  Carboxylic acid metabolism  Cell-cell signaling  Development  Intracellular protein transport  Intracellular transport  Macromolecule biosynthesis  Morphogenesis  Organic acid metabolism  Organogenesis  Phosphoinositide-mediated signaling  Positive regulation of cytosolic calcium ion concentration  Protein biosynthesis  Protein localization  Protein metabolism  Protein secretion  Protein transport  Secretion  tRNA aminoacylation  tRNA aminoacylation for protein translation  tRNA metabolism  tRNA modification  Translation |
